# Editorial: Sorghum and pearl millet as climate resilient crops for food and nutrition security, volume II

**DOI:** 10.3389/fpls.2023.1170103

**Published:** 2023-03-08

**Authors:** Palak Chaturvedi, Mahalingam Govindaraj, Deepmala Sehgal, Wolfram Weckwerth

**Affiliations:** ^1^ Molecular Systems Biology Lab (MOSYS), Department of Functional and Evolutionary Ecology, Faculty of Life Sciences, University of Vienna, Vienna, Austria; ^2^ HarvestPlus, Alliance of Bioversity International and the International Center for Tropical Agriculture (CIAT), Cali, Colombia; ^3^ Syngenta, Jealott’s Hill International Research Centre, Bracknell, United Kingdom; ^4^ Vienna Metabolomics Center, University of Vienna, Vienna, Austria

**Keywords:** climate-resilient, genetic resources, drought tolerance, heat tolerance, disease resistance, biotic stress, abiotic stress, cereals

Achieving global food and nutritional security in the 21st century is challenging as it coincides with climate change and economic vulnerabilities. The reliance on major food crops, i.e. wheat, rice and maize, contributing to 60% of plant-based calories in the entire human diet, further threatens food security. Therefore, it is of utmost importance to explore more native crops, often called orphan crops, and find alternative solutions to address food security. The Food and Agriculture Organization of the United Nations (FAO) has declared 2023 as the International Year of Millets (IYOM) to coordinate agricultural research across continents to scale up the production of alternative nutritious crops and foods. Sorghum and millets are gluten-free, nutrient-rich food crops with a low glycemic index (GI). These grow in marginal areas prone to drought where water-intensive major crops sometimes fail to grow. Sorghum is an important cereal crop grown in south Asia and Africa; it belongs to the same family of Poaceae as millets. Global millet initiatives and biofortification networks are working towards reaching one billion people with biofortified crops by 2030. The current research topic on Sorghum and Pearl millet as Climate Resilient Crops for Food and Nutrition Security, Volume II, comes at the right time to nurture scientific evidence for millet and sorghum research and development, products and promotions. This volume comprises eight manuscripts and aims to provide new insights into the breeding approaches, precision phenotyping, genetic resources, molecular analysis, physiological analysis, biotic stress adaptation mechanisms, nutritional aspects, abiotic stress resistance and gene mapping for the improvement and understanding of the fundamentals of these highly important climate-smart crops.

## Pearl millet

Pearl millet is one of the most produced and consumed cereal crops in Sub-Saharan Africa. This climate-resilient crop is often called a climate-smart crop (Ghatak et al., 2016; Ghatak et al., 2017; Ghatak et al., 2021). During its reproductive phase, it thrives under all adverse environmental conditions such as salinity, drought and extreme heat (Varshney et al., 2017; Chakraborty et al., 2022). Recent evidence has also shown the role of pearl millet in mediating biological nitrification inhibition (BNI) under drought stress (Ghatak et al., 2022). This crop is a multipurpose cereal that provides food and nutrition to undeveloped and developing nations across the globe. In our previous editorial (Chaturvedi et al., 2022) on sorghum and pearl millet as climate-resilient crops for food and nutrition security, we have provided an overview of how these crops can help fight the climate crisis and feed almost 10 billion people by 2050.

Today, Pearl millet is considered one of the important crops to face global warming. Flowering time (FT) is one key trait that could be modulated to face future global changes. In the study by Faye et al., where West African 109 pearl millet landraces accessions were grouped into 66 early-flowering (EF) and 43 late-flowering (LF) varieties, the main aim was to detect genes linked to flowering in general and for traits within each flowering group. The phenotypic diversity and the genomic data of these landraces provided evidence of the role of PhyC gene in flowering in addition to other genes such as FSR12 and HAC1. HAC1 and two other genes (FSR12, Hd16) were in QTLs identified on chromosome 2. In another study, iron (Fe) and zinc (Zn) contents and their combined abilities in West African Pearl millet lines were evaluated (Gaoh et al.). This study selected eight diverse collections based on grain Fe and Zn content, based on agronomic and morphological traits, two landraces and six open-pollinated varieties evaluated. A highly positive correlation was found between grain Fe content and grain Zn content in parental lines and hybrids, suggesting these traits could be improved simultaneously in a variety. The hybrid ICMV 167006 × HKP (2.3 t/ha) was identified as the highest-yielding and most stable hybrid across environments by the GGE biplot (Gaoh et al.). Moreover, enhancing the Fe and Zn content in food grains through plant breeding is a sustainable and economical solution to tackle micronutrient deficiency (approach so-called biofortification) in Asia and Africa, where the resources are very less. For this, three CMS lines along with ten restorers (testers) of pearl millet were used with 31 hybrids to assess the genetic potential to increase the bioavailability of Fe/Zn content. The manifestation of heterosis in F1 provided various cross-combinations that may be guided to enhance the population’s Fe/Zn profile by combining ability tests. A plant population with enhanced Fe/Zn bioavailability, higher grain yields, higher grain Fe/Zn content, and lower phytate content can be designed by delaying cross-combination selection until later generations (Thribhuvan et al.). Many studies indicated that optimizing phytates in the seed is more appropriate than significantly reducing phytate levels, as most plant phosphorous is stored as phytate, which is essential for root growth and development.

Pearl millet is commercially underutilized due to the rapid onset of hydrolytic rancidity of seed lipids post-milling, here the authors have investigated the underlying molecular and biochemical mechanisms of rancidity development in the flour from inbred lines of contrasting genotypes under accelerated ageing conditions (Aher et al.). In this study, the authors have reported the breakdown of storage lipids (triacylglycerols; TAG) and free fatty acid accumulation over the time course for 12 inbred lines, out of which the high rancidity lines had the highest amount of FFA in 21 days, suggesting that TAG lipases may be the cause of rancidity (Aher et al.). Substantial amounts of volatile aldehyde compounds were observed in the high rancidity lines, which are characteristic products of lipid oxidation. Lipases expressed in seed post-milling were sequenced from low and high rancidity lines. Polymorphisms were identified in two TAG lipase genes (*PgTAGLip1* and *PgTAGLip2*) from the low rancidity line. Therefore, these genetic variations can be exploited through marker-assisted breeding or precision genome editing technologies (CRISPR-based approaches) to develop pearl millet elite cultivars with improved flour shelf life creating profits for smallholder farmers and improved markets in South Asia and Sub-Saharan Africa (Aher et al.).

Pearl millet is a unique cereal which can withstand prolonged drought and heat stress. Aquaporin (AQP) genes actively involve desiccation tolerance and water transport. Their potential role in abiotic stress tolerance was functionally validated and systematically characterized (Reddy et al.). This study identifies 34 AQP genes in pearl millet, which are grouped into four subfamilies, including 11 PIPs (plasma membrane intrinsic proteins), 9 TIPs (tonoplast intrinsic proteins), 11 NIPs (nodulin-26-like intrinsic proteins), and 3 SIPs (small basic intrinsic proteins). Phylogenetic relationship within the subfamilies of *PgAQPs* and between AQPs of related species shows their evolutionary relationship and possible functions with their localizations, mainly in the plasma membrane.

Further sequence analysis revealed that PgAQPs are similar to the AQP genes in sorghum. The PgAQPs were expressed differentially under high VPD (vapour pressure deficit) and progressive drought stresses where the PgPIP2 expression was observed; under high VPD and drought stress, six genes showed significant expression (Reddy et al.). Transgenics exhibited better tolerance due to lower Tr (transpiration rate) when compared to WT plants under heat and drought stress. Constitutive expression of pearl millet aquaporin PgPIP2; 6 in tobacco seemed to impart abiotic stress tolerance with reduced transpiration, thereby assisting in water conservation. Tissue-specific or stress-inducible promoters for more specific AQP expression levels can further improve stress tolerance. This study has shown that the molecular breeding of pearl millet is now possible, especially for generating abiotic stress-tolerant cultivars (Reddy et al.). The major biotic stress in pearl millet is the blast or leaf spot disease caused by the ascomycete fungus *Magnaporthe grisea* (Herbert) Barr. This has recently become an economic concern in India (Singh et al.). The present study explains through RNA-Seq and investigates the transcript dynamics in the inbred ICMB 93333 genotype, which had a unique differential reaction to two isolates—Pg 45 (avirulent) and Pg 174 (virulent) of *M. grisea*. The transcriptome results identified 2,300 differentially expressed genes (DEGs) which showed activation or repression of specific genes in various pathways consisting of cell wall defense, pathogenesis-related proteins (PR), primary and secondary metabolic pathways, reactive oxygen species and signalling pathways, which were identified by comparing the host-plant compatible and incompatible interactions. These genes will be searched for their functional SNPs and help develop tolerant variety through marker-assisted breeding (Singh et al.).

## Foxtail millet

In foxtail millet, downy mildew is caused by *Sclerospora graminicola*; leaf cracking and yellowing symptoms develop throughout the leaf surface when infected by this parasite. To identify this pathogenic disease, authors explored the effects on chlorophyll synthesis and photosynthesis of leaves infected by *S. graminicola*. An elite foxtail millet variety, JG21, sensitive to *S. graminicola*, was used. *S. graminicola* inhibited chlorophyll synthesis and caused loose mesophyll cell arrangement (Zhang et al.). Carotenoid content, chlorophyll, net photosynthetic rate (Pn) of leaves and stomatal conductance in infected leaves decreased significantly, and intercellular CO_2_ contents increased. One thousand six hundred eighteen DEGs were detected at five different stages of plant growth. Photosynthesis and light reaction-associated genes were enriched. Based on the weighted gene co-expression network analysis (WGCNA) with 19 gene co-expression modules related to photosynthesis revealed, six hub genes related to chlorophyll synthesis were found suppressed during infection. Weak chlorophyll synthesis and rapid chloroplasts disappearance in foxtail millet was observed succeeding the infection of *S. graminicola*, defence responses and resistance of foxtail millet to *S. graminicola* were inhibited, and the sexual reproduction in *S. graminicola* could be completed rapidly (Zhang et al.).

## Sorghum

Sorghum is a C_4_ photosynthetic, climate-resilient crop producing grain and fodder in harsh environmental conditions (Hao et al., 2021; Prasad et al., 2021; Chaturvedi et al., 2022). It performs best under temperature and water constraints (Griebel et al., 2019). Sorghum is a staple food for more than 200 million people in Africa and Asia; productivity of sorghum in India is low due to shoot flies, drought and grain mold; therefore, in this study, authors have checked the genetic progress made over 30 years and assessed the steps that should be taken to facilitate future genetic improvement (Nagesh Kumar et al.). To achieve the goal, 24 high yielding varieties with better nutritional characteristics and tolerance to shoot fly and grain mold were evaluated in the Telangana state field of India. The study reports a high overall genetic gain of 44.93 kg/ha/yr and 331.63 kg/ha/yr for grain yield and fodder yield, respectively, over the first released sorghum variety CSV 15. The study revealed that sorghum genotypes bred with diverse genetic and physiological backgrounds, such as landraces and with tolerance to pests and diseases, had stable yield performance (Nagesh Kumar et al.).

The above contributions of the research ideas and implementation to this research topic led to valuable cues for a better understanding of sorghum and millets withstanding the climate crisis. These results can facilitate successful breeding for sorghum and pearl millet in the near future. To better understand these crops, combined efforts among upstream research, variety/genotype improvement, and field management are required (Weckwerth et al., 2020; Sanjana Reddy et al., 2021; Ghatak et al., 2023). Finally, we greatly appreciate and sincerely thank the efforts of the journal editors, peer reviewers, and authors. This volume would not have been possible without their significant efforts and contribution. We hope our readers can identify valuable information from this volume and find appropriate collaborators to conduct and promote their great success.

## Author contributions

PC and WW drafted the editorial. MG contributed and provided inputs at the designing stage of the Research Topic. PC, MG, DS, and WW reviewed and critically revised the editorial. All authors read and approved the final version for submission.
